# Supplementary material for: Selection and Evaluation of Porcine circovirus (PCV) 2d Vaccine Strains to Protect against Currently Prevalent PCV2
Source: Vaccines (Basel). 2023 Sep 1;11(9):1447. doi: 10.3390/vaccines11091447 (PMC10534819; doi:10.3390/vaccines11091447)

Figure S1. Phylogenetic analysis and amino acid sequence alignment of PCV2d. Phylogenetic analysis and sequence alignment of 234 PCV2d were conducted by MEGA7.0.21 and CLC workbench, respectively.

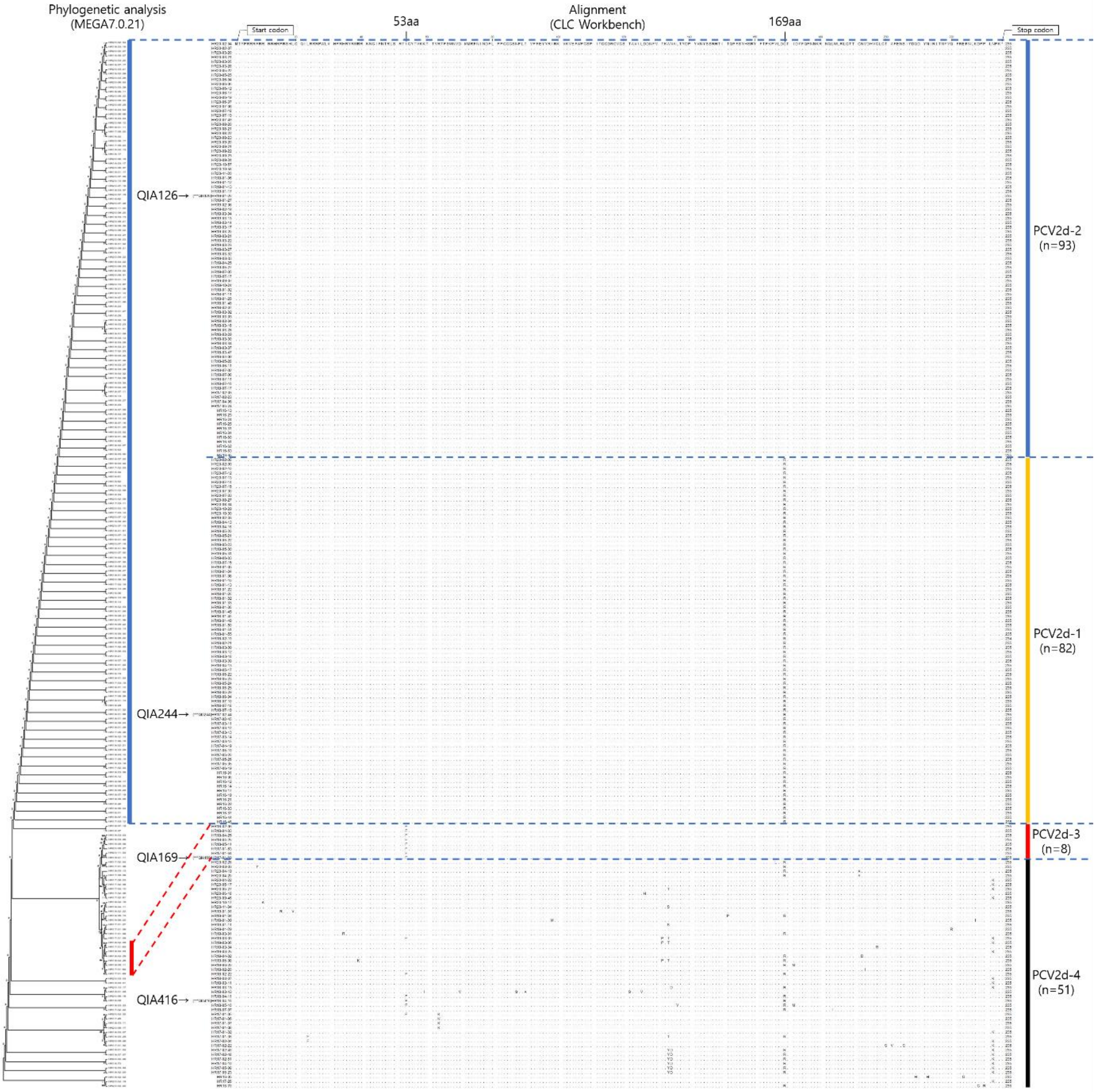

Figure S2. Body weight.

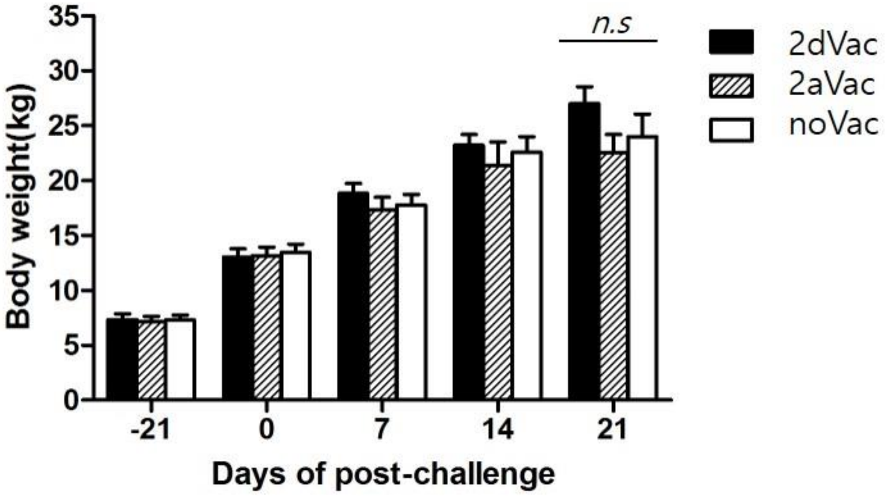

Figure S3. Macroscopic lesions of 2dVac, 2aVac and noVac at 21dpc.

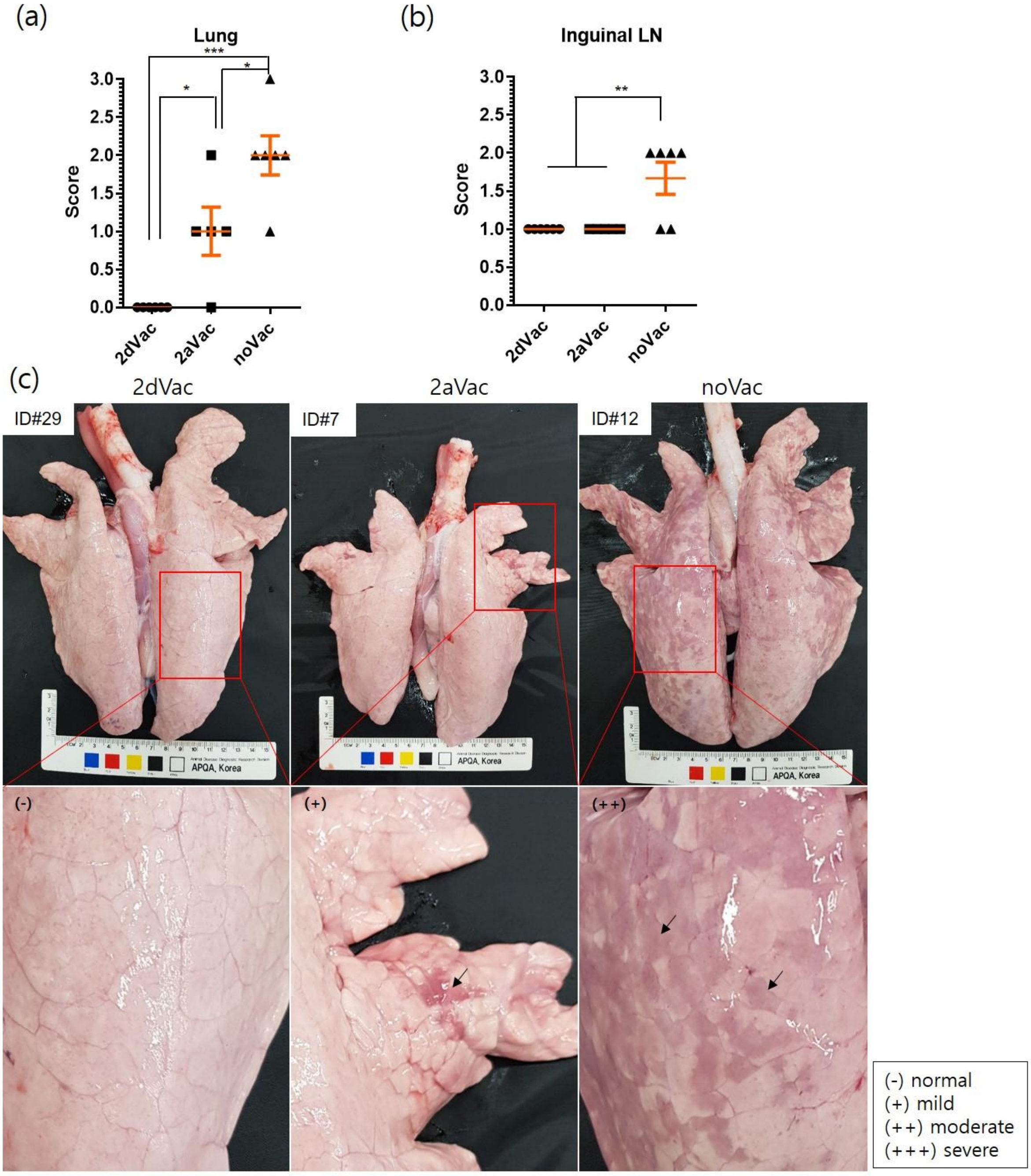

Figure S4. Microscopic lesions of 2dVac, 2aVac and noVac at 21dpc.

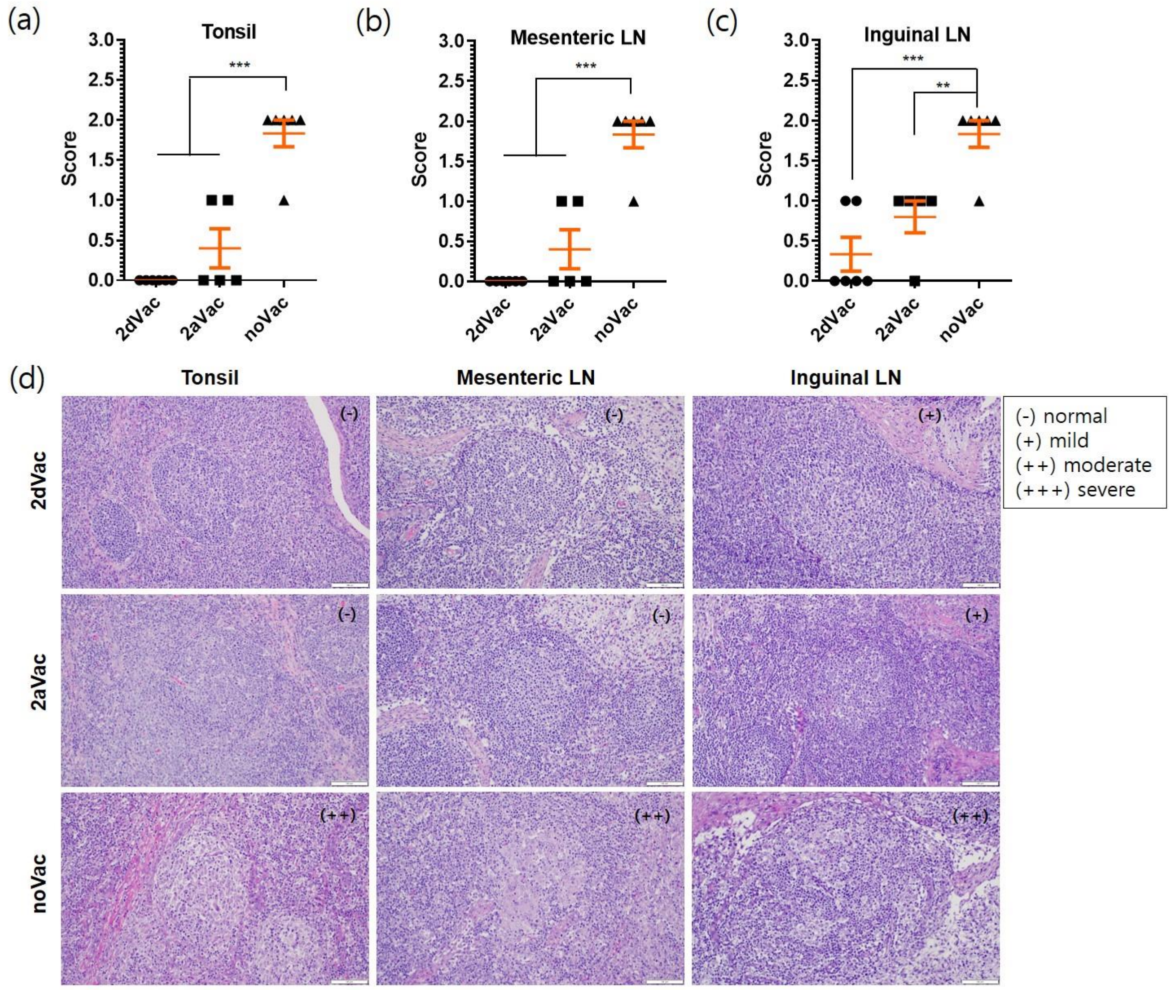

Supplement: Supplementary file 1 [file vaccines-11-01447-s001.zip › vaccines-2568737-supplementary.pdf]
